# Prevalence of dysmenorrhea and predictors of its pain intensity among Palestinian female university students

**DOI:** 10.1186/s12905-018-0516-1

**Published:** 2018-01-15

**Authors:** Heba A. Abu Helwa, Areen A. Mitaeb, Suha Al-Hamshri, Waleed M. Sweileh

**Affiliations:** 1 College of Medicine and Health Sciences, An-Najah National University, Nablus, 44839 Palestine; 2 Department of Community and Family Medicine, College of Medicine and Health Sciences, An-Najah National University, Nablus, 44839 Palestine; 3 Department of Physiology, Pharmacology, and Toxicology, College of Medicine and Health Sciences, An-Najah National University, Nablus, 44839 Palestine

**Keywords:** Dysmenorrhea, University students, Palestine

## Abstract

**Background:**

Few studies on gynaecological problems of young females in Arab countries were published. The aim of this study was to determine the prevalence of dysmenorrhea and predictors of its pain among university students in Palestine.

**Methods:**

A cross – sectional study was carried out on a randomly selected sample of An-Najah National University female students. A pre-designed questionnaire, which included questions regarding menstrual cycle, pain during menstruation, life style, nutritional habits, and symptoms associated with menstrual pain, was used. Pain intensity was measured using visual analogue scale.

**Results:**

A total of 956 female students were involved in the study. Of the total study sample, 846 (85.1%) reported having pain during menstruation; i.e. dysmenorrhea. Dysmenorrhea was significantly [(*p* = 0.027); OR = 1.5, 95% CI (1.05–2.19)] associated with age at menarche. The mean score of pain among dysmenorrhic females was 6.79 ± 2.64. The majority (654; 80.34%) of dysmenorrhic females reported having moderate/ severe pain. Univariate analysis using Chi-square test for factors associated with moderate/severe pain among dysmenorrhic females were irregular cycle [(*p* = 0.015); OR = 1.57, 95% CI = (1.09–2.30)], skipping breakfast [(*p* < 0.001); OR = 1.93, 95% CI = (1.33–2.79)], academic specialization [(*p* = 0.03; OR = 2.2, 95% CI = (1.21–3.98)] for medical specialization with reference to students in humanities), high stress level [(*p* = 0.036; OR = 1.53, 95% CI = (1.03–2.28)], and living in dormitories [(*p* = 0.034); OR = 1.72, 95% CI = (1.04–2.86)]. Multivariate analysis using binary logistic regression enter method showed that medical specialization [(*p* = 0.045); OR = 1.92, 95% CI = (1.02–3.64)] for medical students with reference to students in humanities), skipping breakfast [(*p* = 0.001); OR = 1.96, 95% CI = (1.35–2.86)], and irregular cycle [(*p* = 0.022); OR = 1.56, 95% CI = (1.07–2.29)] were the only significant predictors of moderate/severe dysmenorrhic pain.

**Conclusion:**

There is a high proportion of dysmenorrhea among Palestinian female university students. Skipping breakfast was the strongest predictor for moderate/severe dysmenorrhea. Increased awareness regarding factors that might influence the intensity of dysmenorrhic pain is needed.

## Background

Gynaecological problems among young females in Arab countries are not commonly studied due to social and cultural factors, which consider these problems a prohibited subject. For example, during the time interval from 2012 to 2016, only six articles about dysmenorrhea published in Arab countries were retrieved from Scopus database [1–6]. There has been an ongoing debate on the accurate definition of dysmenorrhea and that is one reason why the prevalence of dysmenorrhea across various studies varies considerably [7]. For example, two recently published studies considered dysmenorrhea as moderate to extreme pain during menstruation while others considered dysmenorrhea as having at least one episode of extreme pain during menstrual cycle [8–10]. However, the bulk of literature on prevalence of dysmenorrhea defined dysmenorrhea as painful menstrual uterine cramps regardless of severity [11–16]. A recent study on dysmenorrhea showed that there are different prevalence rates among females in different countries and different associated factors with dysmenorrhea or severity of pain [17].

Dysmenorrhea is broadly defined as pain during menstruation [18]. Most common symptoms of dysmenorrhea are pelvic or lower abdominal pain, back pain, diarrhoea, or nausea. These symptoms usually start at the time of menstruation and lasts within three days. Terms such as menstrual cramps or painful periods are also commonly used to describe these symptoms [19, 20]. Dysmenorrhea has a negative impact on the quality of life of affected females. For example, dysmenorrhea might negatively affect relationships, academic and professional performance, and social and recreational activities [20]. Dysmenorrhea is classified as either primary or secondary dysmenorrhea. Primary dysmenorrhea usually occurs in adolescence shortly after menarche and is defined as painful menstruation without underlying macroscopic pelvic pathology [21]. Overproduction of uterine prostaglandins is the primary pathogenesis for primary dysmenorrhea [22]. Based on this pathogenesis, the most common medications used to relieve dysmenorrhic pain is non-steroidal anti-inflammatory drugs such as ibuprofen and diclofenac sodium [23]. Secondary dysmenorrhea, is mostly due to an identifiable pathological condition such as endometriosis or pelvic inflammatory disease. The onset of secondary dysmenorrhea usually occur after several years of menarche [21]. Reported risk factors for dysmenorrhea include earlier age at menarche, longer menstrual periods, heavier menstrual flow, and family history of dysmenorrhea [20].

Dysmenorrhea is an important public health problem among various ethnic groups and is associated with loss of school days and poor quality of life [24–26]. In Palestine, a small Arab country in the Middle East, published studies in women’s health in general and in gynaecological problems in particular are scarce. Studies about prevalence of dysmenorrhea and factors associated with it among university female students in Palestine is missing. Therefore, this study was undertaken to establish the prevalence and factors associated with dysmenorrhea among female university students in Palestine. The importance of this study is not only because it is the first in Palestine but also because the Palestinian females have been through a lot of psychological stress due to political stability and insecurity.

## Methods

### Study design and study settings

This was a cross sectional study carried out at An-Najah National University, the largest university in Palestine with more than 10,000 female students distributed across different specialization. This study was carried out during the fall semester of 2016 and included female students from different specialities.

### The tool used (questionnaire) and data collection

The researchers reviewed several articles published among female university students in the Middle East region and developed a questionnaire to be used for the purpose of this survey study. The questions developed in the questionnaire were similar to those found in previously published reports [9, 27, 28]. The language of the questionnaire was Arabic and was written in a very simple straightforward language. A statement regarding the presence of any diagnosed pelvic pathological conditions was stated at the beginning of the questionnaire and students with such pathology were asked not to answer the questionnaire and return it back to the investigators. Therefore, those who filled the questionnaire were students with no pelvic pathological problems and pain due to menstruation is mostly a primary dysmenorrhea.

The questionnaire included 30 questions grouped into three sets of questions. The first set of questions were questions pertaining to reproductive health and life such as age at menarche, length of menstruation, regularity of the menstrual cycle, presence of pain and accompanied symptoms, pain management, life style questions including nutritional habits, place of living, degree of stress, physical activity, and questions pertaining to body weight. The second set of questions included a list of symptoms that could be experienced by females with dysmenorrhea and the participants were asked to choose the list of symptoms that they experience. The third set of questions consisted of visual analogue scale for the student to choose a number representing most close painful feeling during dysmenorrhea. No validation of the visual analogue scale was carried out and therefore the pain intensity reported by the participants remained an approximation of reality rather than an accurate measure. This approximation of pain intensity using the visual analogue scale was used to assess dysmenorrhic pain in several published articles [29–32]. The questionnaire took an average of 10 min to answer all the questions and all the questions asked pertained to the last six months. A copy of the questionnaire (Arabic language) is available upon request from the corresponding author.

The questionnaires were distributed to female students at the university break time from 12 to 1 pm. The questionnaires were distributed in seven different sites in the university where students from all colleges usually spend their break time. The distribution and collection process were carried out for four consecutive days with 300 questionnaires being distributed daily. The distribution and collection of the questionnaires were carried out by two co-authors (H.H and A.M).

### Sample size

The researchers estimated the sample size using Raosoft calculator assuming a response rate of 50% and a confidence level of 95%. The estimated sample size was 380, however, the researchers decided to distribute a minimum of 1200 questionnaire to account for any un-expected scenarios of rejection to participate.

### Ethical approval

The questionnaire included no questions that involve name of the student to ensure confidentiality. Furthermore, an approval was obtained from the institutional review board (IRB) at An-Najah National University was obtained to carry out the study among the female university students. Since no clinical intervention or blood testing was involved in the study, verbal consent, and not a written consent, was obtained from all participants according to IRB regulations.

### Statistical analysis

All data collected were entered in the Statistical Package for Social Sciences (SPSS) 20 software after coding all answers. The variables were then categorized to facilitate the analysis. Examples of categorization include age (< 20 or ≥20 years), age at menarche (≤ 14 or > 14 years), regular menses (always or “not always”), smoking (yes or no), breakfast (always or sometimes/never), and others as shown in Table 1 in result section. Statistical description for categorical variables include frequency and percentage. For the visual analogue scale, it was scaled from 1 to 10. We considered any score ≤ 4 to be of a mild pain while any score ≥ 5 to be as moderate/sever pain. Univariate analysis for the presence of dysmenorrhea (yes versus no) was carried out with all variables pertaining to demographic, menstrual characteristics, and life style. Univariate analysis was also carried out for dysmenorrhic pain intensity (moderate/severe versus mild). For categorical variables, Chi – square test with a pre-set significance value of ≤0.05 was used to test for significant association with moderate/sever dysmenorrhic pain. The dependent variable was pain severity (mild versus moderate/severe). The independent variables used in the univariate analysis were all categorical variables pertaining to demographics, reproductive health, and life style habits. Multivariate analysis was carried out to find predictors of moderate/severe dysmenorrhic pain. Binary logistic regression was employed using the enter method. The dependent variable was the pain severity (mild versus moderate/severe codes as 0 and 1). The independent variables used in binary logistic regression were all variables with significant *p* values in univariate analysis. All through the analysis whenever a significance was obtained, the odds ratio (OR) and the 95% confidence interval (CI) of the odds ratio was calculated and presented.

**Table 1:** Univariate analysis for presence of dysmenorrhea among the study participants

Variables	Total (%)	Presence of dysmenorrhea		*P* value
		YES (%)	No (%)	
College	956	814 (85.1)	142 (14.9)	0.859
Engineering/Science	216 (22.6)	182 (22.4)	34 (23.9)	
Humanities and social sciences	592 (61.9)	507 (62.3)	85 (59.9)	
M	148 (15.5)	125 (15.4)	23 (16.2)	
Age (years)	19.73 ± 1.5			0.806
< 20	704 (73.6)	600 (73.7)	104 (73.2)	
≥ 20	252 (26.4)	214 (26.3)	38 (26.8)	
Age at menarche (years)				0.027
≤ 14	655 (68.5)	569 (69.9)	86 (60.6)	
> 14	301 (31.5)	245 (30.1)	56 (39.4)	
Regular				0.703
Always	248 (25.9)	213 (26.2)	35 (24.6)	
Not always	708 (74.1)	601 (73.8)	107 (75.4)	
Duration of menses (days)				0.339
≤ 5	510 (53.3)	429 (52.7)	81 (57)	
> 5	446 (46.7)	385 (47.3)	61 (43)	
Education of the mother				0.464
≤ High school	625 (65.4)	536 (65.8)	89 (62.7)	
≥ College	331 (34.6)	278 (34.2)	53 (37.3)	
Education of the father				0.761
≤ High school	496 (51.9)	424 (52.1)	72 (49.3)	
≥ College	460 (48.1)	390 (47.9)	70 (50.7)	
Socioeconomic level				0.628
≤ Average (~ 400 USD)	684 (71.5)	580 (71.3)	104 (73.2)	
> Average	272 (28.5)	234 (28.7)	38 (26.8)	
Smoking				0.274
Yes	158 (16.5)	139 (17.1)	19 (13.4)	
No	798 (83.5)	675 (82.9)	123 (86.6)	
BMI				0.099
< 18.5	104 (11.3)	81 (10.3)	23 (16.9)	
18.5–24.9	694 (75.4)	602 (76.7)	92 (67.6)	
25–29.9	101 (11)	84 (10.7)	17 (12.5)	
> 30	22 (2.4)	18 (2.3)	4 (2.9)	
Breakfast				0.984
Every day	243 (25.4)	207 (25.4)	36 (25.4)	
Sometimes/Never	713 (74.6)	607 (74.6)	106 (74.6)	
Tea/coffee/Nescafe				0.0748
≤ 1 cup	312 (32.6)	264 (32.4)	48 (33.8)	
≥ 2 cup	644 (67.4)	550 (67.6)	94 (66.2)	
Vegetables and fruits				0.804
Never or low	203 (21.3)	172 (21.2)	31 (22)	
Moderate	594 (62.3)	506 (62.3)	88 (62.4)	
High	119 (12.5)	104 (12.8)	15 (10.6)	
Very high	37 (3.9)	30 (3.7)	7 (5)	
Meat and protein rich diet				0.328
Never or low	227 (23.8)	190 (23.4)	37 (26.2)	
Moderate	596 (62.5)	517 (63.6)	79 (56)	
High	99 (10.4)	80 (9.8)	19 (13.5)	
Very high	32 (3.4)	26 (3.2)	6 (4.3)	
Sweets and sugary beverages				0.240
Never or low	81 (8.5)	63 (7.7)	18 (12.9)	
Moderate	523 (54.9)	452 (55.6)	71 (50.7)	
High	290 (30.4)	248 (s)	42 (30)	
Very high	59 (6.2)	50 (6.2)	9 (6.4)	
Salty foods				0.326
Never or low	96 (10.1)	77 (9.5)	19 (13.6)	
Moderate	444 (46.6)	387 (47.7)	57 (40.7)	
High	319 (33.5)	269 (33.1)	50 (35.7)	
Very high	93 (9.8)	79 (9.7)	14 (10)	
Exercise				0.721
Yes	329 (34.4)	282 (34.6)	47 (33.1)	
No	627 (65.6)	532 (65.4)	95 (66.9)	
Interval between cycles (days)				0.215
Normal (21–35)	745 (77.9)	640 (78.6)	105 (73.9)	
Abnormal (< 21 or > 35)	211 (22.1)	174 (21.4)	37 (26.1)	
Living				0.866
With family	780 (81.6)	664 (81.7)	116 (82.3)	
In dormitories	174 (18.2)	149 (18.3)	25 (17.7)	

## Results

A total of 956 questionnaires were returned to the researchers giving a response rate of approximately 80% which was higher than we expected. None of the students returned the questionnaire stating a diagnosed pathological condition. Medical students constituted 15.5% of the study sample while the majority (61.9%) were from the faculties of human and social sciences. The mean age of the participants was 19.73 ± 1.5 years and the majority (73.7%) were < 20 years old. More than two thirds (68.5%) of the participants had their first menarche when they were ≤14 years of age. The majority (74.1%) of the participants reported that their menstrual cycle was not regular in the past six months. Regarding the interval between cycles, the majority (77.9%) reported having an interval of 21–35 days between cycles. Two-thirds (65.6%) of the participants reported not doing exercise on their leisure time. The majority (81.6%) of participants were living with their families. Approximately 53.3% of the participants had a duration of menses of ≤5 days. When asked about parents’ level of education, the majority of participants reported that their parents had less than college education. Furthermore, the majority (71.5%) of participants reported that their families’ monthly income is ≤ average Palestinian monthly income (~ 800 USD). A small proportion (16.5%) of the participants was smokers. The majority (75.4%) of participants had normal BMI. Regarding the nutritional practices of the participants, only one forth (25.4%) of the participants reported having their breakfast on daily basis. Approximately two-thirds (67.4%) of the students reported taking ≥ two cups of caffeinated drinks daily. Based on their own assessment, 33.5% reported high consumption of salty foods, 30.4% reported high consumption of sweets, 10.4% reported high consumption of meats, and 12.5% reported high consumption of fruits and vegetables.

A total of 814 (85.1%) females reported having pain during menstruation at least once in the past six months. This percentage is assumingly representing the prevalence of primary dysmenorrhea among the studied sample. The mean score of pain severity as assessed by visual analogue scale among dysmenorrhic students was 6.79 ± 2.64. Univariate analysis for the presence or absence of dysmenorrhea showed that the only significant difference between females with and those without dysmenorrhea was the age at menarche [(*p* = 0.027; OR = 1.5, 95% CI (1.05–2.19)].

Students with dysmenorrhea reported various symptoms that accompany dysmenorrhea, most commonly as fatigue (61.4%) followed by nervousness (40%). Students with dysmenorrhea reported various degrees of pain in the visual analogue scale with 654 (80.34%) having moderate to severe pain and more than half 57.9% reported seeking medications such as paracetamol, NSAIDS, or anti-spasmodic to relieve pain. Regarding site of pain, supra-pubic (77.5%) and back pain (49%) were most frequently reported symptoms. For the duration of pain, the majority reported having pain in the first day of menses only (44.5%). Approximately less than one third (31.1%) reported missing the school in days of menstrual pain. When asked about family history (sister or mother), 73.8% reported a positive history of having painful dysmenorrhea (Table 2).

**Table 2:** Most common symptoms associated with menstruation among female students with dysmenorrhea

Variables	Total number	Percentage
Site of pain		
Suprapubic	631	77.5
Flank	191	23.5
Back	399	49
Thigh	221	27.1
Duration of pain		
Few hours before menses	149	18.3
First day of menses only	362	44.5
From first to third day of menses	288	35.4
More than third day of menses	35	4.3
Severity of pain		
Mild (≤4)	160	19.7
Moderate/severe(> 4)	654	80.3
College absenteeism		
Yes	253	31.1
No	558	68.6
Medications		
Yes	471	57.9
No	338	41.5
Category		
Paracetamol	186	39.5
NSAIDs	219	46.5
Anti-spasmodic	11	2.34
Family		
Yes	601	73.8
No	194	23.8
Associated symptoms		
Diarrhea	95	11.7
Nausea	92	11.3
Vomiting	89	10.9
Fatigue	500	61.4
Dizziness	126	15.5
Headache	148	18.2
Breast tenderness	137	16.8
Nervousness	326	40
Insomnia	131	16.1
Arthralgia	233	28.6
None of the above	38	4.7

Univariate analysis for factors associated with moderate/severe pain among dysmenorrhic females were irregular cycle [(*p* = 0.015); OR = 1.57, 95% CI = (1.09–2.30)], skipping breakfast [(*p* < 0.001); OR = 1.93, 95% CI = (1.33–2.79)], academic specialization [(*p* = 0.03; OR = 2.2, 95% CI = (1.21–3.98)] for medical specialization with reference to students in humanities), high stress level [(*p* = 0.036; OR = 1.53, 95% CI = (1.03–2.28)], and living in dormitories [(*p* = 0.034); OR = 1.72, 95% CI = (1.04–2.86)]. Students in the medical specialization had 2.2 odds of having moderate/severe dysmenorrhea compared to those in the faculties of human and social sciences. Students with irregular menses had 1.57 odds of having moderate/severe menses compared to students with regular menses. Students who reported to have an academic and life stress had 1.53 odds of having moderate/severe dysmenorrhea compared with students who reported no academic stress. Students living in dormitories have 1.72 odds of having moderate/severe dysmenorrhea compared with students living with their own families. Finally, students who do not have regular breakfast had 1.93 odds of having moderate/severe dysmenorrhea compared with students who had breakfast on regular basis (Table 3).

**Table 3:** Univariate analysis for intensity of dysmenorrhic pain

Variables	Total (%)	Mild (%)	Moderate/severe (%)	*P* value	OR 95%(CI)
College					
E	182 (22.4)	36 (19.8)	146 (80.2)	0.03	1.12 (0.74–1.73)
H	507 (62.3)	110 (21.2)	397 (78.3)		Reference
M	125 (15.4)	14 (11.2)	111 (88.8)		2.2 (1.21–3.98)
Age(years)					
< 20	600 (73.7)	115 (19.2)	485 (80.8)	0.556	–
≥ 20	214 (26.3)	45 (21)	169 (79)		
Age at menarche (years)					
≤ 14	569 (69.9)	103 (18.1)	466 (81.9)	0.089	–
> 14	245 (30.1)	57 (23.3)	188 (76.7)		
Regular					
Always	213 (26.2)	54 (25.4)	159 (94.6)	0.015	Reference
Not always	601 (73.8)	106 (17.6)	495 (82.4)		1.57 (1.09–2.30)
Duration of menses (days)					
≤ 5	429 (52.7)	94 (21.9)	335 (78.1)	0.087	–
> 5	385 (47.3)	66 (17.1)	319 (82.9)		
Education of the mother					
≤ High school	536 (65.8)	115 (21.8)	421 (78.5)	0.073	–
≥ College	278 (34.2)	45 (16.2)	233 (83.8)		
Education of the father					
≤ High school	424 (52.1)	87 (20.5)	337 (79.5)	0.518	–
≥ College	390 (47.9)	73 (18.7)	317 (81.3)		
Socioeconomic level					
≤ Average	580 (71.3)	113 (19.5)	467 (80.5)	0.845	–
> Average	234 (28.7)	47 (20.1%)	187 (79.7)		
Smoking					
Yes	139 (17.1)	23 (16.5)	116 (83.5)	0.311	–
No	675 (82.9)	137 (20.3)	538 (79.7)		
Regular exercise					
Yes	282 (34.6)	61 (21.6)	221 (87.4)	0.302	–
No	532 (65.4)	99 (18.6)	433 (81.4)		
Stress					
Yes	249 (30.6)	38 (15.3)	211 (84.7)	0.036	1.53 (1.03–2.28)
No	565 (69.4)	122 (21.6)	443 (78.4)		Reference
Living					
With family	664 (81.7)	140 (21.1)	524 (78.9)	0.034	Reference
In dormitory	149 (18.3)	20 (13.4)	129 (80.6)		1.72 (1.04–2.86)
BMI					
< 18.5	79 (10.1)	12 (15.2)	67 (84.8)	0.363	
18.5–24.9	599 (76.9)	119 (19.9)	480 (80.1)		–
25–29.9	84 (10.8)	18 (21.4)	66 (78.6)		
> 30	17 (2.2)	1 (5.9)	629 (80.7)		
Breakfast					
Every day	207 (25.4)	58 (28)	149 (72)	0.001	Reference
Sometimes/never	607 (74.6)	102 (16.8)	505 (83.2)		1.93 (1.33–2.79)
Vegetables and fruits					
Never or low	172 (21.2)	26 (15.1)	146 (84.9)	0.069	
Moderate	506 (62.3)	98 (19.4)	408 (80.6)		–
High	104 (12.8)	25 (24)	79 (76)		
Very high	30 (3.7)	10 (33.3)	20 (66.7)		
Meat and protein rich diet					
Never or low	190 (23.4)	27 (14.2)	163 (85.8)	0.086	
Moderate	517 (63.6)	113 (21.9)	404 (78.1)		–
High	80 (9.8)	13 (16.3)	67 (83.8)		
Very high	26 (3.2)	7 (26.9)	19 (73.1)		
Sweets and sugary beverages					
Never or low	63 (7.7)	16 (25.4)	47 (74.6)	0.352	
Moderate	452 (55.6)	87 (19.2)	365 (80.8)		–
High	248 (30.5)	50 (20.2)	198 (79.8)		
Very high	50 (6.2)	6 (12)	44 (88)		
Salty foods					
Never or low	77 (9.5)	12 (15.6)	65 (84.4)	0.735	
Moderate	387 (47.7)	80 (20.7)	307 (79.3)		–
High	269 (33.1)	54 (20.1)	215 (79.9)		
Very high	79 (9.7)	14 (17.7)	65 (82.3)		
Tea/coffee/Nescafe					
≤ 1 cup	264 (32.4)	49 (18.6)	215 (81.4)	0.589	
≥ 2 cup	550 (67.6)	111 (20.2)	439 (79.8)		–

Multivariate analysis of variables that showed significance in univariate analysis indicated that medical specialization [(*p* = 0.045); OR = 1.92, 95% CI = (1.02–3.64)], skipping breakfast [(*p* = 0.001); OR = 1.96, 95% CI = (1.35–2.86)], and irregular cycle [(*p* = 0.022); OR = 1.56, 95% CI = (1.07–2.29)] were the only significant predictors of moderate/severe dysmenorrhic pain (Table 4).

**Table 4:** Multivariate analysis for predictors of dysmenorrhic pain severity

Variables	Unstandardised Coefficients (B)	Standardised Coefficients (Beta)	*P* value	95% CI for B
Academic specialization				
Human and social Science	Reference	–	–	–
Medicine and Health Sciences	0.65	1.92	0.045	1.02–3.64
Science and Engineering	0.15	1.56	0.504	0.75–1.78
Regular menses				
Yes	Reference	–	–	–
No	0.45	1.560	0.022	1.07–2.29
Breakfast				
Yes	Reference	–	–	–
No	0.68	1.965	0.001	1.35–2.86
Stress				
Yes	Reference	–	–	–
No	0.39	1.481	0.059	0.99–2.23
Living				
With family	Reference	–	–	–
In dormitories	0.34	1.403	0.228	0.81–2.43

Medical specialization is a weak but significant predictor of moderate/severe dysmenorrhea with an odds ratio of 1.92 (1.02–3.64) with reference to students in the faculties of human and social sciences. The second predictor was period regularity with students having irregular period had higher odds (1.65; 95% CI (1.07–2.29)) of having moderate/severe dysmenorrhea with reference to students with regular periods. The final predictor was having breakfast on daily basis where students who skipped breakfast tend to have higher odds (1.97; 95% CI (1.35–2.86) of having moderate/severe dysmenorrhea with reference to students who had their breakfast on daily basis.

## Discussion

In this study, we aimed to determine the prevalence of dysmenorrhea and predictors of dysmenorrhic pain severity among female university students. The results of our study showed that the prevalence of dysmenorrhea was relatively high (85.1%) but fits within the reported values from developing and developed countries. Published studies showed variable rates of dysmenorrhea ranging from 34% in Egypt, 80% in Australia, 85% among hispanic female adolescents, and 94% in Oman [33–35]. Furthermore, studies showed that the prevalence of severe dysmenorrhic pain varies from 0.9% reported from Korea to 59.8% reported from Bangladesh [33]. It is difficult to explain and interpret variations in prevalence of dysmenorrhea. However, the use of different criteria for the definition of dysmenorrhea in different studies, culture, life style, genetics, and degree of social and personal stress are all potential reasons for variations in prevalence of dysmenorrhea [7, 36–41].

There are several studies on prevalence of dysmenorrhea among female students published from Arab Middle Eastern countries. A cross-sectional study conducted in Dammam University (Kingdom of Saudi Arabia) revealed that about 35% of university females had severe dysmenorrhea [42]. A study among female medical students in Kingdom of Saudi Arabia showed that the prevalence of dysmenorrhea was 60.9%. An Egyptian study reported very high prevalence (94%) of dysmenorrhea among nursing students [43]. A study from Lebanon reported prevalence of dysmenorrhea of 74.3% [44]. Collectively, these studies confirm the variable nature of prevalence of dysmenorrhea among various female students in different Arab countries and even within the same country.

In our study, the most common symptoms associated with dysmenorrhea were physical fatigue and emotional instability manifested as nervousness/irritability. Furthermore, our study showed that approximately 58% of dysmenorrhic students with moderate/severe pain opt to medications to decrease symptoms of painful menstruation. Similar results were obtained in a study published from Iran [45]. In Saudi Arabia, a study conducted among medical female students, the most common symptom that accompanied dysmenorrhea were depressed mood and anger [28]. A study in India indicated that a small proportion of dysmenorrhic students sought pharmacological management of the menstrual pain while the majority used herbal and other non-pharmacological approaches to decrease pain [46]. Seeking for medications or herbal remedies is a common feature among dysmenorrhic female students across most published studies. However, neither in our study nor in most published studies did the young females report seeking for medical intervention to treat dysmenorrhea.

The predictors of dysmenorrhic pain severity had some overlap with the results published elsewhere. In our study, having breakfast was the strongest predictor of intensity of dysmenorrhic pain. The relationship between breakfast and intensity of pain during menstruation have been discussed by an article published from japan where the authors found that students who skip breakfast tends to have higher intensity of dysmenorrhic pain than those who had breakfast on regular basis [40]. Skipping food for cosmetic purposes during adolescence seems to have long term negative effects on reproductive function in young women [47]. Our study showed no relationship between BMI and dysmenorrhea or severity of pain. Similarly, no relationship was found between physical activity, smoking, consumption of salty or sweet food and dysmenorrhea or its pain intensity. Similar results were obtained by a study conducted in Nigeria where researchers reported no significant relationship between dysmenorrhea or its pain intensity with factors such as BMI, waist circumference, and physical activity [48]. A contradictory effect of physical activity on dysmenrrhea was found by a study in Iran [49]. A comprehensive review on the role of physical activity in reducing dysmenorrhea and its pain intensity found little effect but further studies on this issue are needed [50]. Our study showed that the academic specialization was a significant, although weak, predictor of the intensity of dysmenorrhea pain. Several studies were carried out about dysmenorrhea among medical female students since they are under a lot of academic pressure and have to attend the hospital at difficult times. Most of these studies reported high prevalence of dysmenorrhea among medical female students [4, 51–55].

Our study, although the first in Palestine has few limitations that need to be listed. This study is a cross sectional one which limits the causality interpretation. Second, the sample size was large but was obtained from one university in North of Palestine. Larger studies that include young females from all regions in Palestine are needed. Some of the variables in the study, such as nutrition and physical activity ones, might not be well defined and overlap of answers is a possibility which limits the interpretation of data. The definition of dysmenorrhea used in this study, which is any painful menstruation in the past six months, might not be an accurate one. Students with mild pain might be considered by others as normal menstrual cramping. Despite these limitations, our study could be used to increase awareness among female students regarding dysmenorrhea in general and predictors of dysmenorrhic pain severity in particular.

## Conclusion

High proportion of female university female students had dysmenorrhea and more than half of dysmenorrhic females had moderate/sever pain and tend to skip academic classes because of painful menstruation. Among the various nutritional and life style factors investigated, skipping breakfast was the strongest predictor of severity of dysmenorrhic pain. Furthermore, the role of academic specialization as well as irregularity of menses as predictors of severity of dysmenorrhic pain need to be further investigated.

## References

[1] Abd El-Mawgod MM, Alshaibany AS, Al-Anazi AM (2016). Epidemiology of dysmenorrhea among secondary-school students in northern Saudi Arabia. J Egypt Public Health Assoc.

[2] Abdul-Razzak KK, Obeidat BA, Al-Farras MI, Dauod AS (2014). Vitamin D and PTH status among adolescent and young females with severe dysmenorrhea. J Pediatr Adolesc Gynecol.

[3] Potur DC, Bilgin NC, Komurcu N (2014). Prevalence of dysmenorrhea in university students in Turkey: effect on daily activities and evaluation of different pain management methods. Pain Manag Nurs.

[4] Ibrahim NK, Alghamdi MS, Al-Shaibani AN, Alamri FA, Alharbi HA, Al-Jadani AK, Alfaidi RA (2015). Dysmenorrhea among female medical students in king abdulaziz university: prevalence, predictors and outcome. Pak J Med Sci.

[5] Mohamed EM (2012). Epidemiology of dysmenorrhea among adolescent students in Assiut city, Egypt. Life Science Journal.

[6] Mukattash TL, Tahaineh L, AlRawi N, Jarab A, Hammad H, Nuseir K (2013). Behaviors and attitudes towards dysmenorrhea; a crosssectional survey of 2,000 Jordanian university students. Jordan Medical Journal.

[7] De Sanctis V, Soliman A, Bernasconi S, Bianchin L, Bona G, Bozzola M, Buzi F, De Sanctis C, Tonini G, Rigon F. Definition and self-reported pain intensity in adolescents with dysmenorrhea: A debate report. Rivista Italiana di Medicina dell’Adolescenza. 2016;14(2)

[8] Jarrett M, Heitkemper MM, Shaver JF (1995). Symptoms and self-care strategies in women with and without dysmenorrhea. Health care for women international.

[9] Tavallaee M, Joffres MR, Corber SJ, Bayanzadeh M, Rad MM (2011). The prevalence of menstrual pain and associated risk factors among Iranian women. J Obstet Gynaecol Res.

[10] Harlow SD, Park M (1996). A longitudinal study of risk factors for the occurrence, duration and severity of menstrual cramps in a cohort of college women. BJOG Int J Obstet Gynaecol.

[11] El-Gilany AH, Badawi K, El-Fedawy S (2005). Epidemiology of dysmenorrhoea among adolescent students in Mansoura. Egypt. East Mediterr Health J.

[12] Aktas D (2015). Prevalence and factors affecting dysmenorrhea in female university students: effect on general comfort level. Pain Manag Nurs.

[13] Habibi N, Huang MS, Gan WY, Zulida R, Safavi SM (2015). Prevalence of primary dysmenorrhea and factors associated with its intensity among undergraduate students: a cross-sectional study. Pain Manag Nurs.

[14] Hailemeskel S, Demissie A, Assefa N (2016). Primary dysmenorrhea magnitude, associated risk factors, and its effect on academic performance: evidence from female university students in Ethiopia. Int J Womens Health.

[15] Gebeyehu MB, Mekuria AB, Tefera YG, Andarge DA, Debay YB, Bejiga GS, Gebresillassie BM. Prevalence, impact, and management practice of dysmenorrhea among University of Gondar Students, Northwestern Ethiopia: a cross-sectional study. Int J Reprod Med. 2017;2017:3208276. 10.1155/2017/3208276 PMC5446888 28589173

[16] Midilli TS, Yasar E, Baysal E (2015). Dysmenorrhea characteristics of female students of health school and affecting factors and their knowledge and use of complementary and alternative medicine methods. Holist Nurs Pract.

[17] De Sanctis V, Soliman AT, Elsedfy H, Soliman NA, Soliman R, El Kholy M (2017). Dysmenorrhea in adolescents and young adults: a review in different country. Acta Biomed.

[18] Osayande AS, Mehulic S (2014). Diagnosis and initial management of dysmenorrhea. Am Fam Physician.

[19] De Sanctis V, Soliman A, Bernasconi S, Bianchin L, Bona G, Bozzola M, Buzi F, De Sanctis C, Tonini G, Rigon F (2015). Primary dysmenorrhea in adolescents: prevalence, impact and recent knowledge. Pediatr Endocrinol Rev.

[20] Iacovides S, Avidon I, Baker FC (2015). What we know about primary dysmenorrhea today: a critical review. Hum Reprod Update.

[21] Proctor M, Farquhar C (2006). Diagnosis and management of dysmenorrhoea. BMJ (Clinical research ed).

[22] Dawood MY. Dysmenorrhea and prostaglandins. In: Gynecologic endocrinology. Boston: Springer; 1987. p. 405–21.

[23] Zahradnik HP, Hanjalic-Beck A, Groth K (2010). Nonsteroidal anti-inflammatory drugs and hormonal contraceptives for pain relief from dysmenorrhea: a review. Contraception.

[24] Wong LP, Khoo EM (2010). Dysmenorrhea in a multiethnic population of adolescent Asian girls. Int J Gynecol Obstet.

[25] Ahuja A, Sharma MK, Singh A. Impact of dysmenorrhea on quality of life of adolescent girls of Chandigarh. Journal of Child and Adolescent Behavior. 2016;

[26] Unsal A, Ayranci U, Tozun M, Arslan G, Calik E (2010). Prevalence of dysmenorrhea and its effect on quality of life among a group of female university students. Ups J Med Sci.

[27] Al-Jefout M, Seham A-F, Jameel H, Randa A-Q, Luscombe G (2015). Dysmenorrhea: prevalence and impact on quality of life among young adult jordanian females. J Pediatr Adolesc Gynecol.

[28] Chuamoor K, Kaewmanee K, Tanmahasamut P (2012). Dysmenorrhea among Siriraj nurses; prevalence, quality of life, and knowledge of management. J Med Assoc Thai.

[29] Amiri Farahani ËL, Hasanpoor-Azghdy SB, Kasraei H, Heidari T (2017). Comparison of the effect of honey and mefenamic acid on the severity of pain in women with primary dysmenorrhea. Arch Gynecol Obstet.

[30] Gao L, Jia C, Zhang H, Ma C (2017). Wenjing decoction (herbal medicine) for the treatment of primary dysmenorrhea: a systematic review and meta-analysis. Arch Gynecol Obstet.

[31] Kamel DM, Tantawy SA, Abdelsamea GA (2017). Experience of dysmenorrhea among a group of physical therapy students from Cairo University: an exploratory study. J Pain Res.

[32] Mirabi P, Namdari M, Alamolhoda S, Mojab F (2017). The effect of Melissa Officinalis extract on the severity of primary dysmenorrhea. Iranian Journal of Pharmaceutical Research.

[33] De Sanctis V, Soliman AT, Elsedfy H, Soliman NA, Elalaily R, el Kholy M. Dysmenorrhea in adolescents and young adults: a review in different countries. Acta Biomed. 2016;87(1) PMC10521891 28112688

[34] Hillen TI, Grbavac SL, Johnston PJ, Straton JA, Keogh JM (1999). Primary dysmenorrhea in young western Australian women: prevalence, impact, and knowledge of treatment. J Adolesc Health.

[35] Banikarim C, Chacko MR, Kelder SH (2000). Prevalence and impact of dysmenorrhea on Hispanic female adolescents. Arch Pediatr Adolesc Med.

[36] Wang L, Wang X, Wang W, Chen C, Ronnennberg A, Guang W, Huang A, Fang Z, Zang T, Xu X (2004). Stress and dysmenorrhoea: a population based prospective study. Occup Environ Med.

[37] Balbi C, Musone R, Menditto A, Di Prisco L, Cassese E, D’Ajello M, Ambrosio D, Cardone A (2000). Influence of menstrual factors and dietary habits on menstrual pain in adolescence age. Eur J Obstet Gynecol Reprod Biol.

[38] Blakey H, Chisholm C, Dear F, Harris B, Hartwell R, Daley A, Jolly K (2010). Is exercise associated with primary dysmenorrhoea in young women?. BJOG Int J Obstet Gynaecol.

[39] Fujiwara T, Sato N, Awaji H, Sakamoto H, Nakata R (2009). Skipping breakfast adversely affects menstrual disorders in young college students. Int J Food Sci Nutr.

[40] Fujiwara T (2003). Skipping breakfast is associated with dysmenorrhea in young women in Japan. Int J Food Sci Nutr.

[41] Eittah HFA. Effect of breakfast skipping on young females menstruation. Health Sci J. 2014;

[42] Al-Dabal BK, Koura MR, Al-Sowielem LS, Barayan SS (2014). Dysmenorrhea and associated risk factors among university students in Eastern Province of Saudi Arabia. World Family Med J: Incorporating Middle East J Family Med.

[43] El-Hameed NA, Mohamed MS, Ahmed NH, Ahmed ER (2011). Assessment of dysmenorrhea and menstrual hygiene practices among adolescent girls in some nursing schools at EL-Minia governorate, Egypt. J American Sci.

[44] Santina T, Wehbe N, Ziade F (2012). Exploring dysmenorrhoea and menstrual experiences among Lebanese female adolescents. East Mediterr Health J.

[45] Rakhshaee Z (2014). A cross-sectional study of primary dysmenorrhea among students at a university: prevalence, impact and of associated symptoms. Annual Research & Review in Biology.

[46] Omidvar S, Bakouei F, Amiri FN, Begum K (2016). Primary dysmenorrhea and menstrual symptoms in Indian female students: prevalence, impact and management. Global journal of health science.

[47] Fujiwara T (2007). Diet during adolescence is a trigger for subsequent development of dysmenorrhea in young women. Int J Food Sci Nutr.

[48] Maruf FA, Ezenwafor NV, Moroof SO, Adeniyi AF, Okoye EC (2013). Physical activity level and adiposity: are they associated with primary dysmenorrhea in school adolescents?. Afr J Reprod Health.

[49] Mahvash N, Eidy A, Mehdi K, Zahra MT, Mani M, Shahla H (2012). The effect of physical activity on primary dysmenorrhea of female university students. World Applied Sciences Journal.

[50] Daley AJ (2008). Exercise and primary dysmenorrhoea: a comprehensive and critical review of the literature. Sports Med.

[51] Charu S, Amita R, Sujoy R, Thomas GA (2012). 'Menstrual characteristics' and 'prevalence and effects of dysmenorrhea' on quality of life of medical students. International Journal of Collaborative Research on Internal Medicine and Public Health.

[52] Jayanthi B, Anuradha HV (2014). Self-medication practice for dysmenorrhoea in medical, paramedical and non-medical students. International Journal of Pharmaceutical Sciences Review and Research.

[53] Kiran B, Sandozi T, Akila L, Chakraborty A, Meherban RRJ (2012). A study of the prevalence, severity and treatment of dysmenorrhoea in medical and nursing students. Int J Pharm Bio Sci.

[54] Tanmahasamut P, Chawengsettakul S (2012). Dysmenorrhea in siriraj medical students; prevalence, quality of life, and knowledge of management. J Med Assoc Thail.

[55] Yasir S, Kant B, Dar MF (2014). Frequency of dysmenorrhoea, its impact and management strategies adopted by medical students. J Ayub Med Coll Abbottabad.

